# Dielectric Spectroscopy in Biomaterials: Agrophysics

**DOI:** 10.3390/ma9050310

**Published:** 2016-04-27

**Authors:** Dalia El Khaled, Nuria N. Castellano, Jose A. Gázquez, Alberto-Jesus Perea-Moreno, Francisco Manzano-Agugliaro

**Affiliations:** 1 Department Engineering and CIAIMBITAL (Research Center on Mediteranean Intensive Agrosystems and Agrifood Biotechnology), University of Almeria, CEIA3, Almeria 04120, Spain; dalia.elkhaled@gmail.com (D.E.K.); nnovas@ual.es (N.N.C.); jgazquez@ual.es (J.A.G.); 2 Research Group of Electronic Communications and Telemedicine TIC-019, Almeria 04120, Spain; 3 Department of Applied Physics, University of Cordoba, CEIA3, Campus de Rabanales, Córdoba 14071, Spain; aperea@uco.es

**Keywords:** dielectric properties, microwave (MW) application, agrophysics

## Abstract

Being dependent on temperature and frequency, dielectric properties are related to various types of food. Predicting multiple physical characteristics of agri-food products has been the main objective of non-destructive assessment possibilities executed in many studies on horticultural products and food materials. This review manipulates the basic fundamentals of dielectric properties with their concepts and principles. The different factors affecting the behavior of dielectric properties have been dissected, and applications executed on different products seeking the characterization of a diversity of chemical and physical properties are all pointed out and referenced with their conclusions. Throughout the review, a detailed description of the various adopted measurement techniques and the mostly popular equipment are presented. This compiled review serves in coming out with an updated reference for the dielectric properties of spectroscopy that are applied in the agrophysics field.

## 1. Introduction

Concerned with the application of physical methods for the processing of crops and food of agricultural origin, agrophysics is derived from a branch of the agricultural sciences. Agrophysics is a specialized scientific field covering various studies of these material properties and processes [1]. The dielectric assessment of material has been adopted for the electrical characterization of several chemical products and polymer nanocomposites with high abilities of energy storage [2]. For many industrial, food, and agricultural sectors and wood-working branches, material investigation has been used for the evaluation and quality control of meat, fruit, and coffee [1]. A wide spectrum of electromagnetic waves has also been used in moisture measurements. Many authors have based their evaluations on the quality constituents of fruit and vegetables and have succeeded in non-destructive evaluations [3]. Many areas of human activity have taken advantage of the electrical characteristics of agricultural materials [4]. Moreover, producers, handlers, and processors obtain enormous help from the non-destructive techniques in the determination of quality and related characteristics of agricultural products. The interaction of a material with an applied electric field is defined by a dielectric spectroscopy technique. This review focuses on agrophysics and aims to assess the dielectric properties of food material. Dielectric spectroscopy covers an extraordinary spectral range from 10^−6^ to 10^12^ Hz. It is known to be an old experimental tool that has developed dramatically in the last two decades [1]. When water content is high, permittivity and moisture can be closely correlated. In order to determine the rates and uniformity of heating in microwave (MW) thawing, it will be critical to determine the dielectric properties of partially frozen material. In food with pulsed electric fields, ohmic heating, induction heating, and radio frequency (RF) and MW heating, electrical properties are essential in processing. For detection of the processing conditions or the quality of food, these properties are considered essential [5]. Taking into consideration the increased demands of quality and safety, the development of fast and efficient non-destructive methods is imperative. An understanding of the electrical behavior and the development of indirect non-destructive methods to determine the physical properties are both based on the dielectric properties of cereal grains and oilseeds [6].

The basic concepts of electrical properties, the various measuring methodologies, their role in the agri-food sector, and their potential applications in thermal imaging for quality and safety assessment in food processing are a major focus of study in the current research. Looking for correlation dependencies between the dielectric properties and other chemical and physical properties of tested material is the crucial point of study. In fact, taking advantage of the rapid and non-destructive quality assessment of agricultural objects is key in applying the dielectric spectroscopy techniques in agrophysics.

Various characteristics of agricultural materials and food include frost sensitivity, chilling and freezing tolerance, moisture content, seed germination, mechanical stress, pasteurization, and other properties of fruit and vegetables. The properties of seeds, soil, meat, and milk are among these characteristics. Moisture content, the maturity of the fruit, the freshness of the eggs, the potential insect control in seeds, and RF heating are covered by infected food and thus fall within these properties as well.

The interaction of electromagnetic waves with matter, as well as the definition of charge density under an applied electric field, is covered by electrical permittivity [7]. In 1965, Nelson reported the first dielectric properties of grain. For different materials, from solids to liquids and gas, permittivity depends on the dielectric constant and on a loss factor that will be explained in the next section [4].

While the dominant factor is moisture content, other factors can be related to the permittivity of food materials such as the chemical composition, physical structure, frequency, and temperature [8]. The various factors affecting the dielectric property measurements are dissected throughout this review (Section 3).

## 2. Dielectric Properties

### 2.1. Electrical Conductivity

A basic element in ohmic heating is conductivity, where thermal energy is produced from electricity under the flow of an alternating current through food. Regarding its importance in fluid pasteurization, the effective conductivity is essential to learn, next to the overall resistance of liquid particle mixtures. Agricultural materials are assessed by a various number of characteristics including moisture content, maturity, freshness, potential insect control, freezing tolerance, and frost sensitivity. These characteristics can be determined through the conductivity measurement, which is the inverse of the electrical resistivity, which measures the resistance to electric flow. The conductivity measurement measures the electric current flowing through a food material of a unit via the cross-sectional area (*A*), the unit length (*L*), and resistance (*R*). Electrical conductivity (σ) is expressed as follows in Equation (1) [9]:

(1) $$\sigma =\frac{R\times A}{L}$$

The electric conductivity of food has been found to increase linearly with temperature and water/ionic content. The determination of low-temperature characteristics of sensitive vegetables was executed by Maezawa and Akimoto in 1996 [10] using conductivity. By passing a known current of a constant voltage through a known volume of the material and by determining resistance, the conductivity of a material can be settled. The internal structure of agricultural products changes substantially during their deformation [11].

### 2.2. Permittivity and Loss Factor

By definition, the dielectric properties of food are meant to be the electrical properties measuring the interaction of food with electromagnetic fields [12]. In the process of heating, drying, and storage of grains, concerns of relative permittivity, dielectric loss factor, and loss tangent are of high relevance.

A physical quantity describing the dielectric permittivity of a material is symbolized by ε*. This value is given in (Fm^−1^) and expressed in terms of the permittivity of vacuum.

Equation (2) determines the relative permittivity of vacuum (ε_0_), which depends on the speed of light (*c*) and the magnetic constant (μ_0_):

(2) $$c^{2}\mu_{0}\varepsilon_{0}=1$$

The numerical values of ε_0_ and μ_0_ are about 8.854 × 10^−12^ F/m and 1.26 μHm^−1^, respectively.

The permittivity has been recorded as having higher values in other media such as solids, liquids, and gases and has always been expressed relatively to the value of vacuum [13] where ε_abs_ is expressed as Equation (3):

(3) $$\varepsilon_{\text{abs}}=\varepsilon_{r}\varepsilon_{0}$$

This concludes that the relative permittivity ε_r_ of a material can also be expressed as Equation (4):

(4) $$\varepsilon_{r}=\frac{\varepsilon_{\text{abs}}}{\varepsilon_{0}}$$

**Figure 1:**
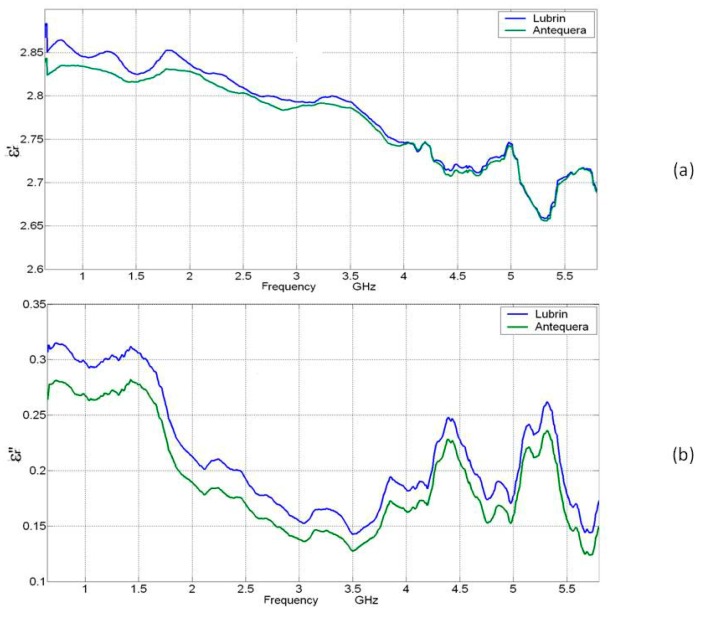
Relative permittivity behavior of the Lubrin and Antequera oil of the “Picual” variety type. (**a**) Dielectric constant (ε’); (**b**) loss factor (ε”).

Both the high frequency and MW fields are sinusoidal time-dependent, which means time-harmonic. Therefore, the permittivity is a complex quantity with real and imaginary components. The equation designating the fundamental electrical property through which the interactions are desired is known as the complex relative permittivity (Equation (5)). An illustrative graph of the relative permittivity (dielectric constant and loss factor) behavior of Lubrin and Antequera oils of the “Picual” variety type is presented in Figure 1.

(5) $$\varepsilon =\varepsilon^{\prime }-j\varepsilon^{\prime \prime }$$

The real part of the dielectric permittivity, known as the dielectric constant, is referred to as ε´ and is related to the capacitance of a substance and its ability to store energy [14]. Due to the applied electric field, the dielectric material stores an amount of energy that is expressed in terms of the dielectric constant (ε´). The imaginary part, known as the loss factor, is referred to as ε” and represents the energy loss of the external electric field when applied to the tested material or whenever the food is subjected to an alternating electric field such as the dielectric relaxation and ionic conduction [5,15]. The loss factor is always positive, has smaller values than the dielectric constants, and is related to various absorption mechanisms of energy dissipations. If the dielectric loss factor is equal to 0, the substance will be lossless [16]. Another descriptive dielectric parameter known as the dissipation factor or loss tangent (tanσ) reflects the ratio of the dielectric loss to the loss factor. This parameter is also used as an index to describe the ability of the material to generate heat [17].

At low frequencies, the direction of the electric field is allowed by the free rotation of the electric dipole of the water molecules. A form of energy storage is presented by this type of polarization. Simultaneously, positive and negative ions of dissolved salts move in accordance with the electric field. Thus, the energy loss will be the result of such an electric current. However, due to the inertia of the water molecules, which is described by the relaxation time (τ), the water molecules will not be able to keep up with the variations in the electric field direction when the frequency rises. This is considered the main reason behind the decrease in the electric field energy storage and the increase in the energy losses. The relaxation frequency of a given polarization mechanism, expressed as (2πτ)^−1^, is the frequency at which this phenomena occurs. Water molecules do not respond to the electric field at higher frequencies.

### 2.3. Polarization of Dielectrics

**Figure 2:**
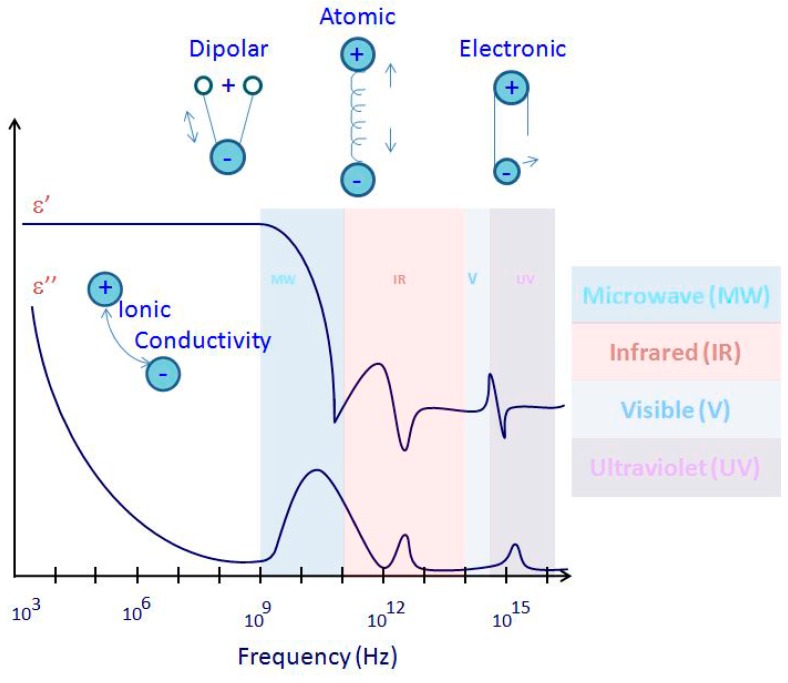
Polarization mechanisms *versus* the frequency range.

An electric dipole is constituted of two charges separated by a distance and is presented by a vector called the dipole moment. Molecules with nonzero permanent electric dipole moments are called polar molecules. Due to a distortion of their electronic distributions and nuclear positions, non-polar molecules can acquire a dipole moment in an electric field. The average dipole moment per unit volume is known as the polarization. A relation between the permittivity and the polarization is given by Equation (6) [13]:

(6) $$P=\left(\varepsilon -1\right)\varepsilon_{0}E$$

where *P* is the polarization, *E* is the electric field, ε − 1 is the electric susceptibility, and ε_0_ is the relative permittivity of vacuum (described earlier). Thus, the relative permittivity is a measure of the polarizing effect from an external field. There are four types of polarization: ionic, orientation, atomic, and electronic polarization [9]. These types are illustrated in Figure 2, ordering the polarization phenomenon occurrence as mechanisms with respect to frequency.

Electronic polarization occurs in atoms where electrons can be displaced with respect to the nucleus. In atomic polarization, the atoms can be moved in crystals or molecules. When they are present together, electronic and atomic polarizations give the material a lossless MW frequency and give the solids a permittivity in the order of less than 10. Atomic or vibration polarization is closely related to electronic polarization. However, the resonant frequencies of atomic polarization are lower due to the greater mass to be moved. Electronic polarization is found to be on the optical band, while atomic polarization is found on the infrared band, as seen in Figure 2 [13]. The dipoles try to follow a rapid changing field in MW or high-frequency fields. Therefore, the energy is lost to the random thermal motion of the water equivalent to a temperature rise known as orientation polarization, where ions move in the direction of the electrical field transferring energy [18,19]. The change accumulation at the surfaces between the materials with different electrical properties (interfacial polarization) and dipole orientation are the major effects behind the polarization.

### 2.4. Power Density and Penetration Depth

The power equation that expresses the rate of heating is presented in Equation (7):

(7) $$P_{v}=2Ef\varepsilon_{0}\varepsilon \prime \prime {\left|E\right|}^{2}$$

where *P*_v_ is the energy developed per unit volume (W/m^3^), *f* is the frequency (Hz), E the electric field, and |*E*| is the electric field strength inside the load (V/m).

The geometry of the load, the oven configuration, and the dielectric properties determine the electric field inside the load. Thus, the determination of the electric field distribution is very complex, making the equation impractical [20,21]. The penetration depth can introduce a better understanding of the dielectric properties.

The distribution of the energy is determined by the transmission properties that are related to the dielectric and thermal properties [17]. The dielectric constant that decreases the speed of propagation makes the wavelength in the dielectric medium shorter than the free space. Thus, a reflection occurs at the interface between two media with different dielectric constants [13,22].

### 2.5. A Sensing Technique

Described as a simple, rapid, and non-destructive measuring technique, much information can be provided about the dielectric response of materials to electromagnetic fields through dielectric properties. This makes dielectric spectroscopy a convenient method for food quality evaluation [23]. In the analysis and monitoring quality of biological materials, dielectric spectroscopy has been used extensively [24]. Because the measurements are rapid and do not destroy the substance, they are suitable for on-line measurements. In agricultural materials and food quality sensing, the utilization of dielectric materials is marked as well.

## 3. Factors, Sensors, and Equipment

### 3.1. Factors Affecting Dielectric Properties

Many years ago, various theoretical studies of the dielectric models of multiphase materials were conducted with verifications [25,26]. Various factors have a great influence on the dielectric properties of food materials. The frequency of the alternating electric field, the temperature, the bulk density, and the moisture content are among these factors [27]. Other listed factors are the ionic nature, the density or concentration, the structure, and the constituents of food materials [28,29,30,31].

#### 3.1.1. Frequency

For moist food, frequency fall causes the loss factor to increase [1]. For constant temperatures, both dielectric constant and loss factor increase with decreasing frequency [32]. Moreover, the penetration depth decreases with temperature, frequency, and moisture content.

For most of the substances, the dielectric permittivity and electrical conductivity are constant only for a limited frequency range. The dielectric permittivity decreases with a frequency rise, while the conductivity suddenly rises. These abrupt changes represent a specific polarization mechanism and are called dispersions. Because of their biological nature, high dispersions are a characterization of agrophysical materials. Due to the interfacial polarization on surfaces between the constituents of a tested material, dispersions at low frequencies are the most common [33]. Today, after the rapid increase in data transfer rates through omnipresent cellular networks, specialized high-frequency electronic components are more available, and measuring devices controlled by computers have become more widespread. Therefore, the measurement techniques of dielectric spectroscopy have undergone a very rapid development, and the possibility of having the necessary tools for MW ranges to validate the developed dielectric models of agrophysical objects has increased.

Reflectometric frequency domain (FDR) and time domain techniques (TDR) have seen an exceptional and prominent technological improvement at high frequencies [34,35]. Due to the relaxation time of free water, estimated to be about 8.3 × 10^−12^ [36], the Gigahertz frequency range is considered essential. The technological development mentioned earlier has increased the capabilities in the MW range, which enlarges the importance of the dielectric spectroscopy techniques in studies of materials more specifically for the properties that change along with variations in water content [1]. When an electrical signal is given, the reflectometric and transmission methods are distinguished among the techniques of the high-frequency domain to analyze the response of a tested material.

#### 3.1.2. Temperature

Knowledge of the dielectric properties of food is highly related to temperature influence. For most foods, electrical conductivity is known to increase linearly with temperature. For granular solids, temperature did not reveal an independence of dielectric properties [37]; they increase dramatically at a moisture content higher than 9.4 GHz [38]. In general, the dielectric constant of fruit and vegetables increases with temperature, but the loss factor can increase or decrease according to the operating frequency [24]. Loss factor mainly increases with temperature [8], and the dielectric constant is higher with low frequencies and starts to decrease at higher frequencies. The temperature dependence of the dielectric constant is minimal in the frequency range between 0.01 and 1.8 GHz [4,39]. The free and bound water content of the food material is considered a major factor in the rate of change of the dielectric constant and loss factor with temperature. The loss factor increases with temperature and with the transition from solid to liquid phase [40,41]. Moreover, the penetration depth decreases with temperature, frequency, and moisture content. However, in the lower moisture range of grapes, a decreasing trend in the loss factor with temperature was observed for low frequencies [42,43].

#### 3.1.3. Molecular Structure

All material in nature possesses dielectric properties that are dependent on their molecular structure—more specifically, on the distribution of electric charges. These charges can be either continuously embedded within the molecules or temporarily induced on their surfaces. Therefore, it can be deduced that dielectric properties can provide a unique identification for a mixture of various molecules. Since the physical and chemical properties of a given material are also determined by the molecular structure of that material, the physical and chemical diversification of a tested material is possible by means of dielectric properties [44].

**Figure 3:**
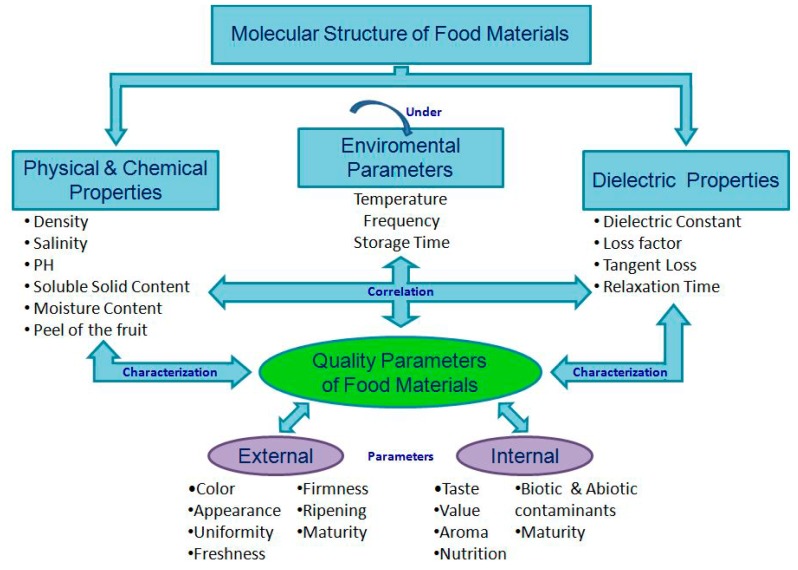
Correlation between dielectric properties and physical/chemical properties.

Because of the unique molecular structure of each material, its behavior will be unique in the electric field. Relating the food products to their commercial and nutritional value, the quality of the material is accessibly determined by its physical and chemical properties. Thus, assuming that the dielectric properties can describe each complex material consisting of various mixed substances can reasonably provide quality information. Figure 3 resumes the systematic assessment of dielectric properties through the correlation with chemical and physical properties under the various environmental parameters where the quality indices vary from one study to another.

A major source in the variation of dielectric properties is the bulk density [45,46,47]. When elaborating functions that determine the grain moisture content, the density dependence of the dielectric properties is a relevant parameter that can be used in the control of the continuous on-line processing of the grain [48]. The main problem in defining the effect of the density is maintaining the composition of the particle size fractions during heating and grinding. The conclusions show that the effect of the particle size is quite negligible compared with the influence of bulk density and other factors.

A simple analyzed example was yoghurt. The necessity of “good yoghurt” is an expression that can be chemically and physically analyzed to effectively receive a set of numbers that uniquely describe this particular sample of yoghurt. Density, viscosity, protein content, fat, water carbon, nitrogen, the amount, and type of bacteria are all properties that can be examined under chemical and physical properties. For better knowledge of the material under consideration, a greater gathering of its data is needed, which would make it possible to replicate the yoghurt sample while conserving its “goodness”. A fast and reliable indicator containing comprehensive information of the “good yoghurt” parameters is necessary. Thus, a quality index describing the uniqueness of a test material would be unambiguously needed for easy and quick measurements. Moreover, it will be more desired in such applications to make non-destructive and non-invasive measurements. Achieving this objective becomes promising with the dielectric spectroscopy of agrophysical objects [44].

The molecular structure of any heterogeneous material is a direct analysis of its quality parameters through the correlation sought between its dielectric properties and its physical and chemical properties (Figure 3). The phase shift, the signal attenuation, relaxation time, and temperature influence in function of frequency are functions relying on dielectric properties that can be correlated with physical and chemical properties such as moisture, firmness, color, pH, salinity, acidity, content of starch, of sugar, aromatic compounds, and trace elements [1].

#### 3.1.4. Water Content

The quality of most of the agricultural origin products is essentially affected by water content and organic salts present in the materials; this is translated in its commercial value [49]. By determining the unique properties of water naturally, it becomes possible to determine the water content in porous materials. This is considered a fundamental and direct application of the dielectric properties in agrophysics. In fact, the assessment of agricultural porous media, including soil, granular, and powder materials of agricultural origin, is based on moisture and salinity.

Due to its degradation susceptibility in the presence of water, grain stored in silos cannot be too moist. Financial losses can occur due to an excessive moisture of grain [50].

Manufacturing processes, transportation, and improper storage are all reasons behind defecting biological materials. Volumetric proportions and concentrations of aqueous solutions present in the objects might be affected. However, the rapid, non-destructive, reliable, and definite assessment of the quality parameters of the porous bodies becomes accessible with the widespread use of dielectric permittivity sensors and meters.

For the production, trade, processing, and storage of these materials, the water content control of porous media has been enabled with the application of MW techniques through the development of non-destructive methods. MW aquametry is the name of the newborn field of metrology [51,52]. In fact, MW techniques have proven to be more advantageous than other methods such as radio frequencies (order of 1 MHz), infrared, or ionizing radiation because of the particular properties of MW radiation (frequencies between 1 and 100 GHz).

Both the dielectric constant and loss factor increase with the increase in moisture content. On the other hand, penetration depth decreases with increases in the temperature, frequency and moisture content [32,53].

#### 3.1.5. Salinity, Fat, and Other Constituents

Salinity strongly affects the quality of agrophysical materials as well. In fact, salinity affects the ionic conductivity of these objects, so it becomes one of the constituents of the imaginary part of the complex dielectric permittivity of agrophysical materials. This can be described by the following equation from Chelkowski [54]:

(8) $$\varepsilon_{r}^{*}=\varepsilon_{r}^{´}-j\varepsilon_{r}^{"}=\varepsilon_{r}^{´}-j(\varepsilon_{D}^{"}+\varepsilon_{\sigma }^{"})$$

where $j=\sqrt{-1}$. This equation attributes the energy losses of the applied electric field to the inertia of the dipole molecules in high frequencies defined as (ε_D_´´) and to the ionic conductivity defined as (ε_σ_´´).

The second component of the energy loss is a result of salt distribution and is described by Equation (9):

(9) $$\varepsilon_{\sigma }^{"}=\frac{\sigma }{2\pi f\varepsilon_{0}}$$

In 1929, Debye developed another mathematical model of the complex dielectric permittivity of polar liquids [1] and is given by Equation (10):

(10) $$\varepsilon_{r}^{*}=\varepsilon_{\infty }+\frac{\varepsilon_{s}-\varepsilon_{\infty }}{1+j\omega \tau }$$

In this equation, $\varepsilon_{\infty }$ represents the value of the real part of the dielectric permittivity of polar molecules at very high frequencies. At very low frequencies, this value is given by ε_S_. ω is the angular frequency (ω = 2π*f*), and τ is the molecule relaxation time.

The experiments studying the frequency dispersion of the real and imaginary part of a NaCl water solution shows that the energy loss is mainly caused by ionic conductivity at low frequencies and by the inertia of the electric dipoles (dipole dielectric loss ε_D_´´) at high frequencies.

Since most food products have a loss factor less than 25, this implies a penetration depth of 0.3 to 1 cm. Unfortunately, the literature data covers only food components or pure foods but did not hold information on complex or formulated foods where the dielectric properties must be measured and estimated [55]. In 1974, Ohlsson and his coworkers [53] proved that the dielectric properties were largely influenced by water and salt content, specifically at 450 and 900 MHz.

The influence of the water, salt, or ash content (amount of minerals present) depends largely on the way they are bound and restricted in their movements by other food components [56,57]. Observations have shown that the addition of salt increased the loss factor and decreased the dielectric constant in different food materials.

For the case of salty food materials, both the dielectric constant and the loss factor of food materials record a remarkable increase with decreasing frequency for constant temperatures. For low MW frequencies, the loss factor generally increases, but it only conserves the same increase pattern at higher MW frequencies for salty food [56].

The loss factor is known to increase with temperatures in materials with high-moisture and low-fat contents [58,59]. Dielectric properties of different food and food products were found to increase with fat and lipid content.

For higher frequency ranges up to 2800 MHz, the increase of dielectric loss with temperature was only noticed for salty food at lower MW frequencies [53,60].

### 3.2. Dielectric Sensors

The choices of measuring technique equipment and sample holder design depend upon the dielectric materials being measured. At a limited frequency range, the cavity perturbation method was extensively used for liquid samples at both low and high temperatures. Solids and liquids at broader frequency ranges can be investigated through reflection-based measurements.

Various scientific studies dealing with the diversification of the quality parameters based on electric and dielectric properties have dealt with this hypothesis on different tested objects [27,61,62]. However, it is still rare to find practical materials characterizations implemented in a broad frequency range by dielectric spectroscopy. Enhancing the dielectric measurements and development of advanced sensors can enlarge the scope of investigation in this field [1,63].

Another major importance is the design of equipment for radiofrequency and MW dielectric heating applications. The applications include grain-frying, seed treatment for the improvement of seed germinations and insect control, and MW electrical fields [63].

### 3.3. Measuring Equipments

Similar to other selections and constructions of test fixtures, dielectric permittivity equipment depends on the examined dielectric material from liquids to solids or mixtures. Besides the texture of the material, other features such as the measurement volume, the frequency range, the required accuracy, and the availability of the equipment, including financial resources, also affect the dielectric measurement [44,64,65]. Impedance analyzers and scalar network analyzers [66] are considered cheaper than vector network analyzers (VNAs), but their applications are limited to the frequency range of the α and β dispersions. For testing a material, researching the dipolar polarization mechanisms (dispersion) is useful for broadband frequency examinations [67]. Although classified as expensive, the versatility of the VNA serves for such measurements.

The Agilent open-coax probe of type 87,070, which is an accessory of the VNA [68], is essentially used in multiple experiments. Although this measuring system is considered to be expensive for industrial applications, it is the only sensor that is available commercially for broadband dielectric property measurements applied to liquid and solid materials to date.

**Figure 4:**
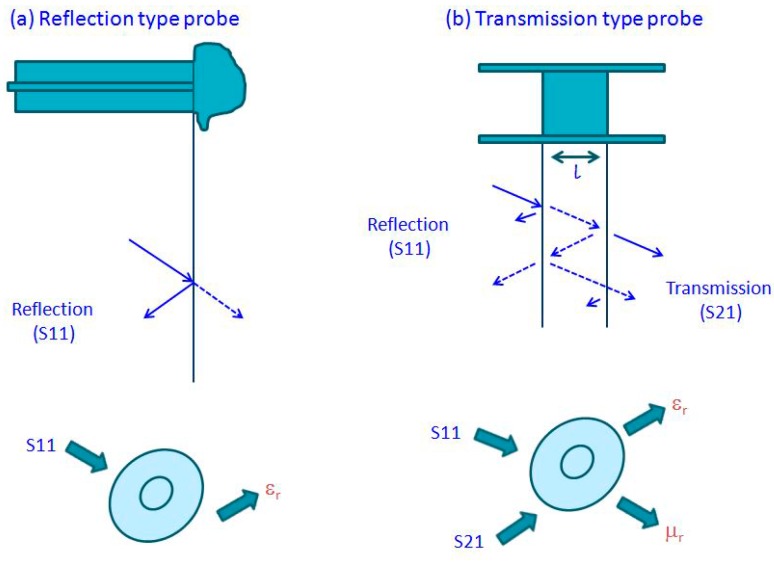
Reflection types of the probe (**a**); and transmission types of the probe (**b**).

For the spectrum of liquids, biological materials and multiphase mixtures, a commonly used instrument for the measurement of the complex dielectric permittivity at the frequency range from 0.2 to 50 GHz is the open-coax probe of type 85,070 from Agilent [4]. With the VNA comes an accessory: a probe with specialized software. The surface of contact between the probe and the tested material can be expressed by the measurement of the S11 reflection parameter. A phase shift is designated by the reflected signal of the probe and the incident signal of the VNA. The ratio of the magnitude change and this phase shift is referred to as the S11. The illustrations of these measurements are shown in Figure 4, which highlights the difference between the reflection and transmission types of probes. For the user to acquire real and imaginary parts of the dielectric permittivity of the material, some typical models are incorporated in the supplied software. Instead of the expensive 85070E probe, some other subminiature version (SMA) coaxial connectors, or a modified type N, can be used for more economical solutions. These can perform correctly up to several GHz frequencies [69,70]. On the other hand, the S21 parameter, which is the ratio of the transmitted signal through the tested material to the incident one, can be measured using coaxial transmission lines or wave waveguides. In fact, the S21 parameter, when interpreted, allows for determinations of the complex dielectric permittivity and the magnetic permeability of a sample (Figure 4) [71,72,73].

The VNA (such as in the case of the Rhode and Schwarz) generates, at the reflective mode of work, a sine wave signal in a broad frequency range from 20 KHz to 8 GHz. At this point, the separation occurs between the incident and the reflected signal [74]. In fact, the dielectric properties of the material determine the phase and the amplitude of the signal reflected from the probe. On the basis of the theoretical models that describe the probe in a dielectric medium, the frequency spectra of the dielectric constant and loss factor ε´(*f*) and ε´´(*f*) can be calculated respectively. For liquids with commonly known ε*(*f*) characteristics [75], some numerical calculations are required in addition to the calibration measurement of the objects. A necessity that must be stressed here is that the application of high-frequency fields is required for the measurement of the real part ε´(*f*) of ε*(*f*). In addition, the elements of the measuring system must be treated as a distributed parameter system [76].

Applying techniques and equipment used in impedance spectroscopy is possible at low-measurement frequencies where the permittivity sensor may be regarded as a lumped parameter [77]. A capacitor with a capacity that changes depending on the soil water content describes sensors used in FDR meters for soil moisture measurements. For soil moisture measurements, instruments performing at a constant frequency below 100 MHz are widely commercially available [78,79]. However, some significant measurement errors due to the salinity of the soil are caused by the fact that these instruments require individual calibrations [75].

For dielectric spectroscopy applications, the reflectometric technique allows for the construction of portable sensors and meters for the assessment of dielectric properties of materials, which is not the case for the transmission technique [44]. For that, the dielectric spectroscopy is considered to be very promising for the industrial quality control of materials and products of agricultural origin [64] and biomass [80]. The materials and products of agricultural origin could be assessed for their quality [81,82]. The quality of the aforementioned materials becomes possible with these techniques, even during their processing in real time.

Fourier transform obtained from a reflectogram of the sensor responding to the forcing pulse can get the frequency spectrum of complex dielectric permittivity. In this case, two or three stainless steel rods placed in a tested material and forming a section of a parallel waveguide can act as a dielectric permittivity sensor. In fact, the telecommunication technology by agrophysics adopts the TDR technique as a means to evaluate soil moistures [83] and soil salinity [84]. The input data of these transforms is obtained by this technique.

Along the waveguide with an input having a length (*l*), an electric pulse travels and reflects from its end and returns. The calculation of the travel time (*t*_p_) at a 2*l* distance can be achieved through the “in time identification” of the pulse inside the waveguide. The following equation implies the appropriate relation [85]:

(11) $$t_{p}=\frac{2l}{v}=\frac{2l}{c}\sqrt{\frac{\varepsilon {´}_{r}}{2}\left[\sqrt{1+\tan^{2}\delta_{e}}+1\right]}$$

where the loss tangent or the dissipation factor (δ_e_) is defined in Equation (12):

(12) $$\delta_{e}=\frac{\varepsilon_{r}\prime \prime }{\varepsilon_{r}\prime }$$

where *v* is the speed of the pulse in the material, and c is the speed of the light in the vacuum. However, it is possible to assume that tan^2^ δe ≅ 0 for the case of a non-saline material with negligible conductance and that, therefore, the formula will be simplified to the following equation:

(13) $$t_{p}≅\frac{2l}{c}\sqrt{\varepsilon_{r}^{\prime }}$$

The length of the parallel waveguide and its salinity affect the measurement frequency range. Among the factors cited on which the frequency range depends are the material frequency dispersion and the rise time of the pulse. However, this range cannot be defined by the real part of the complex dielectric permittivity. Moreover, the case of soil in natural conditions is an example of a measured material with a high probability of not being homogeneous. This is the reason behind referring to the calculated real TDR value of the dielectric permittivity as the name of the apparent bulk or dielectric permittivity. The mean value of a certain ε_r_ material can be presented by placing the TDR sensor parallel waveguide in a cylindrical volume only in the middle. In the determination of water content of porous materials, the resulting value of the dielectric permittivity is approximated over the sample volume determined by the length of the waveguide rod [86,87]. This is considered one of the main advantages of the TDR techniques, especially for the inhomogeneous distribution of water inside the volume of a material such as soil.

In fact, the availability of the equipment makes the TDR techniques very popular, feasible for automatic and non-destructive measurements, and easy for calibration. For diverse material groups, the determination of moisture content is considered practical with this universal form.

This technique has also been used for analyzing the spatial variability of soil moisture [88], assessing the liquid petrochemical materials in industrial applications [89], and determining the soil density [90].

A significant scientific achievement and practical implementation in this area is achieved by the Institute of Agrophysics PAS in Lublin [91]. The Institute of Agrophysics PAS in Lublin has developed TDR meters for salinity, soil moisture, and temperature; these meters are used by laboratories worldwide.

In recent years, two devices have been developed by the Institute of Agrophysics PAS in Lublin and are considered as exemplary implementations of the dielectric spectroscopy.

A telemetric system of the soil moisture, temperature, and salinity is provided to securely access the web server in Lublin and retrieve the stored data.

Long-term research on such topics as the monitoring of flood banks, places endangered by mudslides, and soil water balance determinations, lie within the scope of this system. Other major advantages of this system reside in the functionality and the low power consumption of the measurement.

### 3.4. Microwave Heating

MW heating has been used widely for several applications and has been studied for the pretreatment of biomass materials [92]. When a grain batch is subjected to RF, it becomes possible to selectively destroy the pests that are feeding on grains such as grain weevils; and this is considered as a major MW heating application. At a much higher frequency of the applied electric field, the cereal grains are found to be more susceptible than the biological tissues of pests. The loss factor of wheat weevil has been shown to achieve a maximum value of the RF range from 10 to 100 MHz [4]. For the development of effective pasteurization techniques, heating via an alternative electric field may be used where harmful microorganisms are usually eliminated through the application of pasteurization processes before the packaging of products. To delay the crystallization processes, preserve the commercial properties of food products maximally, and dissolve the sugar crystals, the major process used is thermal conditioning. However, due to the small thermal conductivity, conventional thermal treatments require high temperatures and therefore are time-consuming. The treated products may be negatively affected by destroying the vitamins and nutritional substances for example [93]. By transferring the electromagnetic energy directly to the interior of the material, Wang and his coworkers [94] found that dielectric heating enables the fast heating of a selected volume [95]. The product quality is preserved, and the effectiveness of the pasteurization processes increases due to the bulk influence on the sample, the energy efficiency, and the high rate of RF and MW. Achieving high efficiency of RF and MW heating for a given material undergoing thermal treatment is a necessary condition for penetration depth and the optimal selection of frequency [24]. Moreover, the impact of RF and MW heating efficiency will vary accordingly to the influence of the dielectric properties for each thermally treated material in particular. These properties include the water content, the viscosity, and other chemical composition properties [96].

### 3.5. Measuring Techniques

Due to the non-destructive monitoring of some specific materials properties, the measurement of dielectric properties is gaining more importance. These measurements are finding increasing application as new electro-technology become more adapted for agricultural use and the food-processing industry. Venkatesh and Raghavan [64] have completed a comprehensive overview of the different measuring techniques. In the MW region, the dielectric properties of food can be determined using different MW measuring sensors. Measurement techniques used for the sensing of the properties of materials samples can be categorized either as reflection or transmission types, using resonant or non-resonant systems, with open or closed structures [24]. While the waveguide and coaxial line are under transmission measurement and present closed structures, the free space transmission measurement and open-ended coaxial line system are open-structure techniques. The most practical methods appear to be the ones that utilize the measurement of the dielectric properties and the agri-food products of the grain [97]. These techniques include waveguide measurements, resistivity cell, parallel plate, lumped circuit coaxial probe transmission line, resonant cavity, free space, parallel plate capacitor, cavity resonator, and time domain spectroscopy, each having unique advantages and disadvantages [13,98]. Since they were only used for high loss materials and low frequencies, the lumped circuit techniques are no longer used.

**Figure 5:**
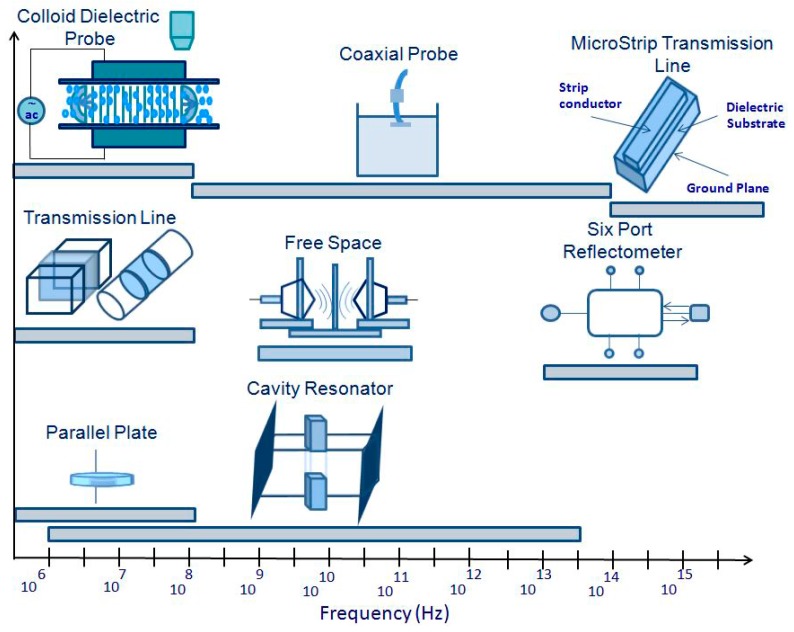
Dielectric properties techniques used for the agri-food characterization *versus* the frequency range.

Open-ended coaxial probe was the most commonly used technique for determining the dielectric properties of high loss liquids and semi-solid foods [99,100], and of fresh fruit and vegetables [101]. Basically, the nature of the dielectric material, the sample physical state (liquid or solid), its shape (thickness, flatness), the electrically desired frequency range, and acquired temperature predetermine the choice of the method for any desired application [102]. Thus, it is upon the dielectric materials, the extent of the research, the available equipment, and the sample holder design that the choice of measurement equipment will be made. The major techniques used for the agri-food sector are illustrated *versus* frequency in Figure 5.

The cavity perturbation technique (Table 1) is commonly used for homogeneous food materials. The design of this technique can either be in the standard TM (transverse magnetic) or TE (transverse electric) mode of propagation of the electro-magnetic fields. Liao and his coworkers [103] have reported the measurement details and the perturbation equations adapted for the calculation of the dielectric constant and the loss factor, along with the accuracy information. The measurement of garlic at selected levels of moisture content was executed using this method at 35 °C to 75 °C.

As a modification to the transmission line method, the coaxial probe method was used (Table 1). The open-ended coaxial probe technique was also used for the measurements of dielectric properties in thermal treatments of controlling insects in fruit at a temperature ranging from 1 to 1800 MHz [59]. This technique is not suitable to execute measurements on granular and pulverized samples with bulk density.

The transmission line method (Table 1) has a narrower range of frequencies than the coaxial probe method but is differentiated for its accuracy and sensitivity. In order to avoid the influence of ionic conductivity and bound water relaxation, such a method is used to determine the moisture content with a frequency above 5 GHz [104]. A swept frequency network analyzer can be used for a more sophisticated implementation of this technique, where the impedance is measured automatically as a function of the frequency. The fact that the sample must be cut into a slab makes it a cumbersome technique [64].

Another technique to determine the permittivity is the use of a MW resonator (Table 1) that can be partly or completely filled with the material. Obtaining the complex impedance spectrum of a resonator is key to this easy and accurate characterization [105]. This technique might not be applicable for gases with low permittivity, but applies to all liquid and solid materials.

Time domain spectroscopy (Table 1), developed in the 1980s, covers a frequency range from 10 MHz to 10 GHz. This technique has been used to measure the dielectric properties of a honey-water mixture, which has been investigated and tabulated at a frequency range from 10 MHz to 10 GHz at 25 °C [106].

Free space transmission (Table 1) is based on two assumptions. First, the uniform plane normally depends on a homogeneous material having a flat surface and the planar sample having an infinite extent [64]. Implementations are easy for industrial applications for continuous monitoring and control, such as the determination of moisture content and the measurement of the density [107].

The transmission line (Table 1) is defined as the thin width to height ratios at least. Micro strips have been used as MW components for a considerable number of years, as they have been deemed suitable for use for dielectric permittivity measurement. This technique allows a relatively straightforward measurement of effective permittivity and implementation in industrial equipment. Determining the effective permittivity of a micro strip is covered by an unknown dielectric substance [108].

Non-destructive broadband permittivity measurements can be provided by a six-port reflectometer technique with an accuracy comparable to commercial instruments. A more complex mathematical procedure to translate the signal characteristics into useful permittivity data is involved in this method [64].

Colloid dielectric probe is a technology that can measure the dielectric properties of colloidal liquid materials. The fields of study include pharmaceutical, biochemical, and food industries [9].

## 4. Applications

The main reason behind the practical interest in determining the dielectric properties of food products and agricultural materials resides mainly in the need to optimize the MW frying and heating processes [1] and develop a faster and more reliable technique for measuring the water content [24]. In 1973, Hasted comprehensively developed the dielectric behavior of single-phase materials such as water and methanol [109]. However, most of the agrophysical material in the study is categorized as a multiphase material like soil, which makes the analytical description of the complex dielectric permittivity quite complicated. The physical and chemical effects that occur on phase boundaries are considered major contribution factors causing this complexity.

These materials can be particularly diversified with respect to their dielectric properties and correlated with quality parameters of the tested objects such as physical and chemical properties (see Figure 3).

Two major agrophysical applications of the dielectric spectroscopy are the measurement of water content in liquids, solids, and other multiphase mixtures and MW heating applications.

During MW cooking or other processes that involve RF or MW dielectric heating, food and agri-food materials are exposed to the electromagnetic fields, and it is essential to understand the permittivity and dielectric properties of food materials.

In the aim of building a successful application of grading based on electromagnetic energy and developing heating processes, it is important to determine the optimum frequency range in which a material has the desired dielectric properties, the basic knowledge needed to determine the frequency and dielectric properties [4]. This selection also aims at the realization of improvements in the design of MW and RF heating equipment [18,110,111]. In fact, the level of interaction between food and high-frequency electromagnetic energy defines these properties, which makes them important for the design of food and meals intended for MW propagation [32,39,112]. The penetration depth achieved in a certain food and the physical characterization of biomaterials can also be evaluated through dielectric properties. Moreover, the investigation of seed treatment improves germination via the suggested dielectric properties [4].

### 4.1. Fruit and Vegetables

The quality parameters of fruit include firmness, soluble solid content, pH, electrical conductivity, and moisture. These properties can be improved by rapid, reliable, and non-destructive assessment, all of which can help in efficient production, harvesting, storage, and processing.

Currently, the research based on the dielectric spectroscopy techniques through its measurement potential to examine fruit and vegetable quality is conducted in various countries; however, the scope of examination is limited to laboratories. The practicality of such techniques is always under further investigation.

The exploration of the dielectric properties of fruit and vegetables by several researchers [113,114,115,116] aims to achieve a better understanding of the interaction between fruit and vegetables, and electromagnetic energy. The use of dielectric property sensing as a potential non-destructive characterization of quality factors shows great potential in sensing peach maturity and chilling injury in sweet potatoes. Few rapid and non-destructive quality measurements for fruit and vegetables have been executed at MW frequencies, and the identification of permittivity-based maturity indices for tree-ripened peaches has been achieved [4]. Table 2 summarizes the experiments. For dried fruits with low water content, the dielectric constant is low as well. Broadband permittivity measurements were initiated to study the dielectric properties of several fruits and vegetables at a frequency range from 200 MHz to 20 GHz [117].

**Figure 6:**
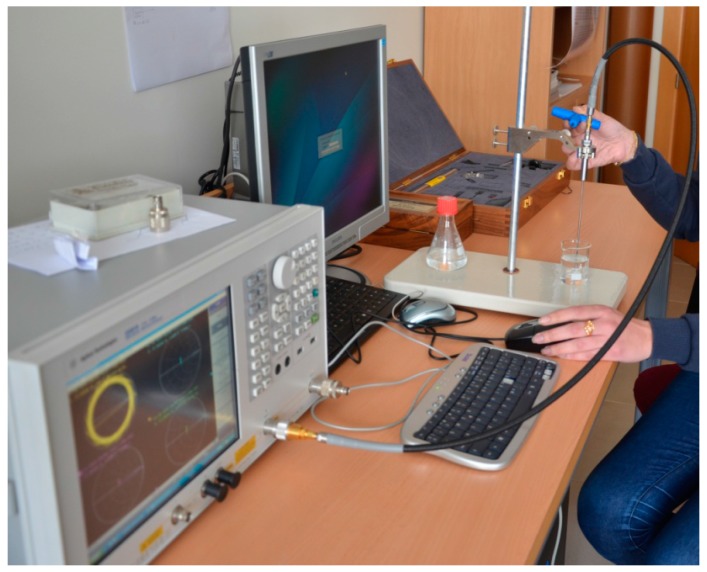
Keysight 85070E dielectric probe used for measurement of dielectric properties.

Figure 6 shows a typical measurement executed using the Keysight 85070E dielectric probe kit.

### 4.2. Granular Materials of Agricultural Origin

Various studies have described the dielectric properties of grains and seeds in a wide range of frequencies and moisture levels [4,117]. The dielectric permittivity of cereal grains as a function of frequency, density, and moisture has been developed through theoretical models [118,119,120,121]. Nelson reported the first dielectric properties of grain 45 years ago, namely, that loss factor decreases with the frequency increase. For moist food tested, in most of the cases, both dielectric constant and loss factor increased significantly as frequency decreased. On the other hand, dielectric properties were increasing sharply with the transition of temperature from −10 °C to 0 °C. Concerning density, an elaboration of functions determining grain moisture content was executed by Venkatesh and Raghavan. The influence of the particle size in heating and grinding were negligible in comparison with bulk density. In this matter, several models for chickpea, green pea, lentil, and soybean flour were executed at moisture contents varying from 8 to 21 g/100 g, at a frequency ranging from 10 to 1800 MHz, and at a temperature ranging from 20 °C to 90 °C, using an impedance analyzer and an open-ended coaxial probe [9]. Dielectric spectroscopy has been shown to have great research potential in this field. Further experiment results are presented in Table 2.

### 4.3. Liquid Materials of Agricultural Origin

Liquid foods and agricultural materials of various groups were examined. At a frequency range from 0.1 to 1 MHz, Lizhi and his coworkers studied the dielectric properties of fatty acids and vegetable oils in 2008 [122]. Further experiment results are presented in Table 2.

### 4.4. Dairy Products

Dairy products that are considered interesting agrophysical objects have complicated chemical compositions. Further experiment results are presented in Table 1.

## 5. Conclusions

As a primary motivation to pursue research on the correlation between dielectric properties and physical/chemical properties, it is essential to determine the quality of agricultural products and food materials so as to meet the consumers’ expectations that are growing quickly. Developing specific and advanced techniques and instrumentations enhances the scope of these investigations, which basically rely on being provided with the appropriate technologies. Investing in a non-destructive characterization technique must be relevant for the environmental and economical scientific assessment in the food industry.

This review covers the fundamentals of the dielectric spectroscopy. The dielectric characterization applications in agrophysics have been collected along with their techniques and measurements. The solid relationship between dielectric behavior and the chemical and physical structure of the agrophysical material in the studies highlighted throughout the review opens the door to explore the existing investigations further and to elicit more accurate indices. With the availability of updated analyzers on the market, future research is encouraged to invest deeply in the dielectric characterization of the agricultural and food sector. The dielectric spectroscopy has been proven to have great potential in the electrical assessment of cereal grains, fruit and vegetables, and various other food industry products. Upon the various factors affecting the dielectric properties such as frequency, moisture content, bulk density, temperature, composition and concentration, structure and constituents, carbohydrate, and both the ash and protein content of food materials, future research is expected to elicit more studies on the correlation between physical and chemical properties and dielectric properties.

## Figures and Tables

**Table 1:** Classification of techniques used for the dielectric properties determination.

Technique	Frequency Range (Hz)	Description	Material	Advantages	Disadvantages
Cavity Perturbation	10^6^ to 10^14^	Based on: The shift in resonant frequency. The change in absorption characteristics of a tuned resonant cavity.	Homogeneous food material. Low dielectric loss materials.	Simplicity of sample preparation. Accuracy. Easily adaptable to low and high temperatures. Capability. Easy data reduction.	Restriction to a single frequency measurement. Necessity of calibration for each cavity.
Open-Ended Coaxial Probe	5 × 10^8^ to 10^11^	Senses the signal reflected. 3.5-mm diameter coaxial line. Probe with flat ranges.	High loss liquids. Semi solid foods. Materials with low values of absolute permittivity. Fresh fruit and vegetables.	Little/No sample preparation. Easy. Convenient. Wide frequency range.	Air gap produced between the sample and the end of the probe. Air bubbles.
Transmission Line	<10^8^	Sample of the substance put inside an enclosed transmission line.	Liquid and viscous fluid types of food.	Accuracy. Sensitivity.	Narrow range difficulty in the preparation of sample.
Resonator	5 × 10^7^ < 10^11^	Partly or completely filled with the material. Material put directly inside the waveguide.	Liquid and solid materials.	Accuracy.	–
Waveguide	10^9^ to 2 × 10^10^	Sample holder useful for measurement	Grain Seed. Fruit and vegetable tissue samples.	–	–
Time Domain Spectroscopy	10^7^ to 10^1^^0^	Use of the reflection characteristics of the food material.	Substance homogeneous.	Rapidity. Highly accurate. Wide frequency range. Very small sample size.	Little percent error is recorded. Expensive.
Free Space	10^9^ to 10^11^	Sample placed between two antennas: Transmitting & receiving. Phase shift of the signal and attenuation recorded.	Inhomogeneous material.	No special sample preparation. High temperature. Accuracy. Wide frequency range.	–
Micro strip transmission line	7 × 10^10^ to 10^11^	Defined as thin width to height ratios. Micro strips used as microwave components. Effective permittivity of a micro strip transmission line depends on the permittivity of the upper region.	–	Straightforward measurement	–
Six-port reflectometer	5 × 10^10^ to 7 × 10^10^	Provides non-destructive broadband permittivity. Open-ended coaxial probe immersed on the test liquid at constant temperature.	Liquids.	Automatic data acquisition. Full automatic reduction. Accuracy comparable to commercial instruments wide variety of biomedical applications.	–
Colloid dielectric probe	75 × 10^3^ to 3 × 10^7^	Provides the frequency characteristics *versus* the permittivity.	Colloidal liquid materials.	Rapid and accurate measurements	Polarization effect cause errors due to the ionic materials measured with metal electrodes.

**Table 2:** Experiments executed on various food products for dielectric property determination.

Concept	Product	Reference	Frequency (MHz)	Result
Fruit & Vegetables	Apple	[123]	3–40	Use of coaxial probe.
		[124]	300–900	Variation with maturity and drop with aging.
		[125]	–	Dielectric properties constant during storage time for 14 weeks.
		[126]	–	Dielectric constant decreases with temperature and frequency.
		[127,128]	500	Development of new maturity index based on dipole relaxation frequency. Good correlation of the new defined maturity index and Thiault index.
	Apple juice	[101]	200	Temperature dependence similar to water.
	Apple peels	[129]	–	Dielectric constant decrease with frequency.
	Apple pulp	[74]	–	Linear decrease of permittivity with frequency.
	Carrot	[123]	2–40	Inflection point and critical edge frequency at 100 MHz.
	Cooked peas	[130]	2800	Values recorded ranging between 54 and 65.
	Eggplant	[131]	–	Surface electrical resistance increases quadratically between 14.5 and 1612.6 KΩ.
	Grape	[1]	–	Increase of loss factor with storage time.
	Guava	[1]	–	Increase of permittivity with temperature.
	Macadamia nut kernels	[59]	–	Penetration depth decreases with increase of frequency, temperature and moisture content.
	Mango	[4]	–	RF penetrated deeper in mangoes than MW. Suitable for potential postharvest disinfestation treatment.
	Mashed potatoes	[130]	2800	Values recorded ranging between 61 and 76.
	Melons	[132]	–	Linear dependence of permittivity with soluble solid content (glucose and fructose).
		[113]	10–1800	Estimation of soluble solid content.
	Orange	[1]		Temperature linear increase below 50 MHz.
	Peach	[123]	4–40	Use of coaxial probe.
	Potato	[123]	1–40	Frequency linear decrease.
	Potato starch	[133]	1200–18,000	–
Granular materials	Raw potatoes	[134]	–	Dielectric properties drop with frequency.
	chickpea, lentil, soybean, green pea	[9]	10–1800	–
	Grain	[4]	–	Loss factor decreases with the frequency increase.
Liquid materials	Acetic acid & vinegar	[135]	0.1–1	Dielectric constant decreases linearly with temperature increase.
	Fruit juice	[136]	–	–
	Wine & grape juice	[137]	200–3000	–
Dairy products	Natural yoghurt	[138]	1000–20,000	Detecting sugar concentration.
	UHT milk	[139]	1000–20,000	Chemical species smooth for distinction.

## Abbreviations

The following abbreviations are used in this manuscript:

σ	Electrical Conductivity
ε	Complex Dielectric Permittivity
δ_e_	Dissipation Factor
*A*	Unit Cross Sectional Area
*c*	Speed of light
μ_0_	Magnetic Constant
*E*	Electric field
|*E*|	Electric field strength inside the load
*f*	Frequency
FDR	Reflectometric Frequency Domain
*L*	Unit Length
MW	Microwave
*P*	Polarization
*P*_v_	Energy developed per unit volume
*R*	Resistance
RF	Radio Frequency
SMA	Sub Miniature version A
SPR	Six-port reflectometer
tanσ	Loss Tangent
TDR	Time-Domain Reflectometry
TE	Transverse Electric
TM	Transverse Magnetic
*t*_p_	Travel time
VNA	Vector Network Analyzer
ε’	Dielectric Constant
ε’’	Loss Factor
ε_0_	Relative permittivity of vacuum
ε_abs_	Absolute Permittivity
$\varepsilon_{\infty }$	Dielectric constant at high frequencies
ε_σ_´´	Inertia of Ionic Conductivity
ε_D_´´	Inertia of Dipole molecules
ε_r_	Relative Permittivity
ε_r_*	Relative dielectric permittivity
Ω	Angular frequency
ε_s_	Dielectric constant at low frequencies
τ	Relaxation Time
